# Attenuation of p38-Mediated miR-1/133 Expression Facilitates Myoblast Proliferation during the Early Stage of Muscle Regeneration

**DOI:** 10.1371/journal.pone.0041478

**Published:** 2012-07-24

**Authors:** Duo Zhang, Xihua Li, Chuchu Chen, Yuyin Li, Lei Zhao, Yanyan Jing, Wei Liu, Xiaoyun Wang, Ying Zhang, Hongfeng Xia, Yaning Chang, Xiang Gao, Jun Yan, Hao Ying

**Affiliations:** 1 Key Laboratory of Nutrition and Metabolism, Institute for Nutritional Sciences, Shanghai Institutes for Biological Sciences, Graduate School of the Chinese Academy of Sciences, Chinese Academy of Sciences, Shanghai, China; 2 Department of Neuromuscular Disease, Children’s Hospital of Fudan University, Shanghai, China; 3 School of Biotechnology of East China University of Science & Technology, Shanghai, China; 4 Model Animal Research Center, and MOE Key Laboratory of Model Animals for Disease Study, Nanjing University, Nanjing, China; 5 Zhejiang Provincial Key Lab for Technology and Application of Model Organisms, School of Life Sciences, Wenzhou Medical College, Wenzhou, China; Goethe University, Germany

## Abstract

Myoblast proliferation following myotrauma is regulated by multiple factors including growth factors, signal pathways, transcription factors, and miRNAs. However, the molecular mechanisms underlying the orchestration of these regulatory factors remain unclear. Here we show that p38 signaling is required for miR-1/133a clusters transcription and both p38 activity and miR-1/133 expression are attenuated during the early stage of muscle regeneration in various animal models. Additionally, we show that both miR-1 and miR-133 reduce Cyclin D1 expression and repress myoblast proliferation by inducing G1 phase arrest. Furthermore, we demonstrate that miR-133 inhibits mitotic progression by targeting Sp1, which mediates Cyclin D1 transcription, while miR-1 suppresses G1/S phase transition by targeting Cyclin D1. Finally, we reveal that proproliferative FGF2, which is elevated during muscle regeneration, attenuates p38 signaling and miR-1/133 expression. Taken together, our results suggest that downregulation of p38-mediated miR-1/133 expression by FGF2 and subsequent upregulation of Sp1/Cyclin D1 contribute to the increased myoblast proliferation during the early stage of muscle regeneration.

## Introduction

Skeletal muscle has considerable capacity to regenerate following myotrauma [1], [2]. The first step of regeneration is largely dependent on the activation, cell cycle entry, and proliferation of myogenic satellite cells. The satellite cell population may have clinical applications in the treatment of devastating and deadly diseases such as muscular dystrophy. Although many regulators of proliferation processes have been revealed in recent years, a better understanding of the regulation is still required.

MicroRNAs (miRNA) are ∼22-nucleotide noncoding RNAs that negatively regulate gene expression by promoting mRNA degradation and/or inhibiting mRNA translation through sequence-specific interactions between a seed sequence of miRNA and a seed match sequence located in the 3′-untranslated region (UTR) of the target mRNA [3], [4]. miRNA genes are one of the most abundant classes of regulatory genes in mammals, and mounting evidence indicates that miRNAs are key regulators for diverse cellular processes including differentiation, apoptosis, proliferation, metabolism, immunity, and development [4].

A number of miRNAs, which have been characterized as modulators of myoblast proliferation and myogenic differentiation, are thought to be involved in muscle regeneration as well as myopathies such as muscle dystrophy [5], [6]. Among them, miR-1 and miR-133 are well characterized [5]. A series of studies have clarified that miR-1 promotes the skeletal myoblast differentiation and myocyte fusion [7], [8] and inhibits myoblast proliferation [9]. In contrast, miR-133 promotes skeletal myoblast proliferation [8], while it represses cardiac muscle proliferation [10] and inhibits cardiac hypertrophy [11]. The difference between miR-133 effect on skeletal muscle and cardiac muscle regarding proliferation remains to be further studied. Furthermore, the molecular mechanisms underlying muscle regeneration, especially how upstream signalings influence regulatory miRNAs expression and how myogenic satellite cell proliferation is orchestrated by multiple miRNAs still need to be delineated.

The activation, cell cycle entry, and proliferation of myogenic satellite cells are coordinated by multiple growth factors. It has been reported that extracts from crushed muscles contain mitogenic activities and trigger quiescent satellite cells to enter the cell cycle [12], [13], [14], [15]. FGF2 is potent in recruiting satellite cells to break quiescence and enter the proliferative phase [16], [17]. The release of FGF2 from the monocytes and macrophages as well as damaged myofibers, is proportional to the degree of injury [18], [19], [20]. It has been demonstrated that FGF2 stimulates myogenic satellite cell proliferation.

The signaling pathways that transduce the FGF signaling have recently been investigated with the use of both transgenic techniques and pharmacological inhibitors. These studies revealed that the MAP kinase pathway is important for FGF-induced increase in satellite cell proliferation [21]. In the absence of p38α, myblasts exhibit delayed cell cycle exit and continuous proliferation in differentiation-promoting conditions, indicating that p38 it is a critical regulator of myoblast cell cycle exit, a necessary step prior to commencing the muscle differentiation gene program [22]. It has been reported that p38 signaling mediates miR-1 in hypoxic cardiomyocyes [23]. However, whether p38 signaling is involved in FGF-induced satellite cell proliferation during muscle regeneration and whether p38 signaling is the upstream signaling for miR-1 and miR-133 that might play pivotal roles in myoblast activation and proliferation at early stage of muscle regeneration is currently not clear.

The aim of this work is to explore the miRNAs regulatory network involved in the pathological pathways participated in skeletal muscle regeneration by using several animal models and patient samples. miRNAs upstream signalings, miRNA expression, miRNA target genes, as well as downstream key factors were investigated in muscles undergoing regeneration. In this study, our finding suggests that miR-1, and miR-133, as well as p38 signaling are attenuated in regenerating muscles. Additionally, p38 signaling is required for miR-1/133a clusters transcription. Importantly, both miR-133 and miR-1 are able to induce myoblasts growth arrest at G1 phase. Mechanistically, Sp1 and its direct target gene Cyclin D1 have been identified as novel target genes for miR-133 and miR-1, respectively, which in turn mediate the effect of miR-133 and miR-1 on cell cycle progression. Finally, we also revealed that FGF2, which is elevated during muscle regeneration, represses p38 activity, leading to the downregulation of miR-1 and miR-133 and upregulation of Cyclin D1. Based on our results, we proposed a novel mechanism of FGF2-stimulated myoblast proliferation during muscle regeneration, which involves p38 signaling mediated miR-1/133 transcription and their direct downstream targets Sp1 and Cyclin D1.

## Results

### miR-1/133 Expression and p38 Activity are Decreased during Early Muscle Regeneration

**Figure 1:**
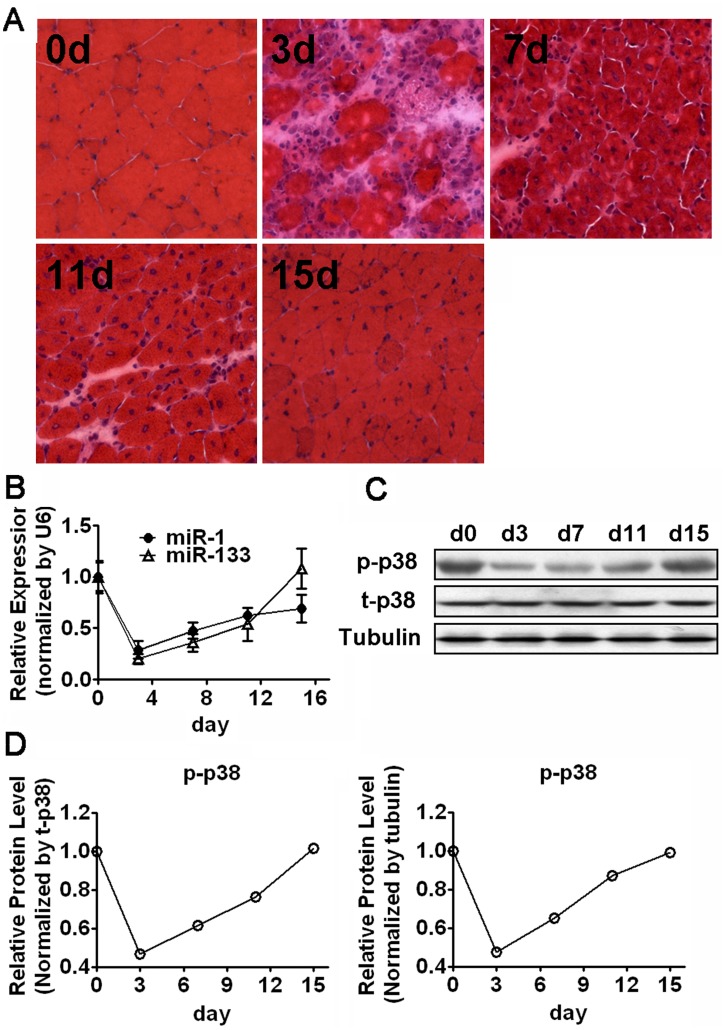
miR-1/133 Expression and p38 Activity are Attenuated in Regenerating Muscle Tissues. (A) Hematoxylin and eosin staining of GAS muscle of mice at the indicated time points following freeze injury. (B) Real-time RT-PCR analysis of the time-course expression of miR-1 and miR-133 in GAS muscle of mice following freeze injury. (C and D) Western blot analysis of phospho-p38 (p-p38) in GAS muscle at the indicated time points following freeze injury. The densities of p-p38 bands were quantitated and normalized to total p38 (t-p38) or tubulin as indicated.

It has been shown that muscle specific miRNAs, miR-1 and miR-133, might play important roles in myogenic proliferation and differentiation, as well as in muscle regeneration and muscular disorder [5], [24], [25], [26], [27]. However, the expression pattern and function of miR-1 and miR-133 have never been studied in freeze injury or lidocaine injury models (Figure S1A). We determined the expression of miR-1/133 in gastrocnemius (GAS) muscles at 7 days following freeze injury or lidocaine injection as described in Materials and Methods. Consistent with the results reported in cardiotoxin-treated regeneration model, the expression level of miR-1/133 was significantly decreased after muscle damage (Figure S1B) [9]. We then investigated the expression of miR-1/133 at different time points during muscle regeneration in freeze injury model (Figure 1A and Figure S1C). A significant decline in miR-1/133 expression followed by a later recovery was observed (Figure 1B). These results suggest that the downregulation of miR-1/133 expression is associated with muscle regeneration and might be important for the myoblast proliferation during the early stages of muscle regeneration. Concomitant with the downregulation of miR-1/133 expression, we found p38 activity was also decreased quickly and significantly after freeze injury in the regenerating muscle tissues (Figure 1C, D).

### p38 Signaling is Required for the Transcription of miR-1/133a Clusters

**Figure 2:**
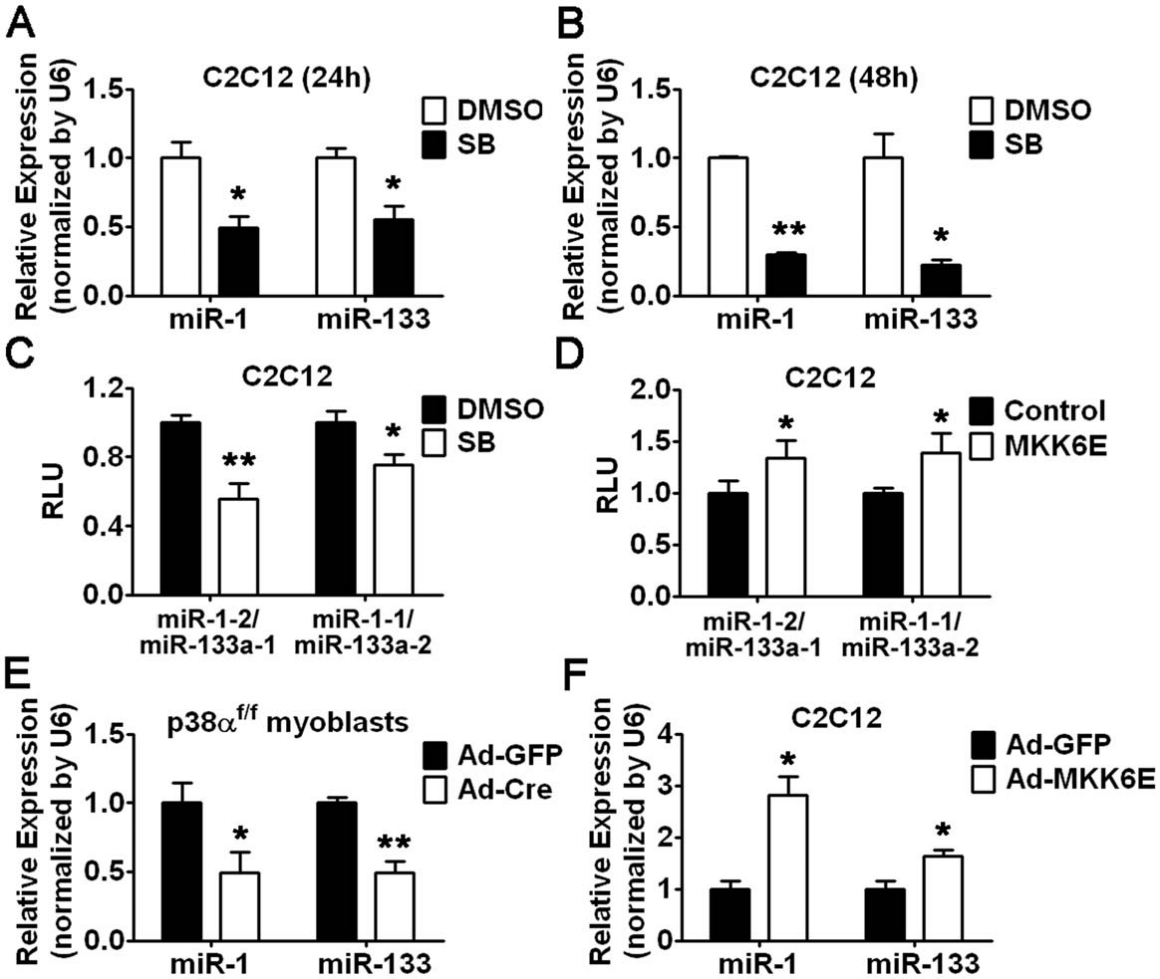
p38 Activity is Required for miR-1/133a Cluster Transcription. (A and B) Real-time RT-PCR analysis of miR-1/133 expression in C2C12 myoblasts treated with SB203580 for 24 hours (A) or 48 hours (B) as indicated. (C and D) Enhancer activity of miR-1–2/miR-133a-1 or miR-1–1/miR-133a-2 cluster in C2C12 myoblasts treated with SB203580 for 24 hours (C) or infected with MKK6E (D). (E and F) Real-time RT-PCR analysis of miR-1/133 expression in p38α^f/f^ myoblasts infected with Ad-Cre or Ad-GFP as a control (E) or in C2C12 myoblasts infected with adenoviral MKK6E (Ad-MKK6E) or Ad-GFP (F). Error bars represent the SD of three independent experiments. *p<0.05, **p<0.01.

Although miR-1 and miR-133 have been studied during muscle regeneration, the upstream signalings and detailed mechanisms remain to be further clarified. As previously reported, p38 activity is required for modulating cell cycle exit and myogenesis [22], [28]. Based on our finding that both the expression of miR-1/133 and the activity of p38 were attenuated at the early stages of muscle regeneration, we speculated that p38 signaling might be one of the upstream signalings regulating miR-1/133 expression. To test our hypothesis, we investigated the relationship between p38 activity and the transcription of miR-1/133 *in vitro*. Interestingly, we found the expression of miR-1/133 was gradually reduced in C2C12 myoblasts treated with SB203580, an inhibitor of p38α and p38β (Figure 2A, B), indicating that the activity of p38 is required for the transcription of miR-1/133. Since miR-1 and miR-133 have several family members, which are located on different chromosomes as clusters with each other or with miR-206, to identify which family members are regulated by p38 activity, we determined the SB203580 effect on the transcription of miR-1–2/miR-133a-1, miR-1–1/miR-133a-2, and miR-133b in C2C12 myoblasts. As shown in Figure 2C, SB203580 only suppressed the activity of the enhancers of miR-1–2/miR-133a-1 and miR-1–1/miR-133a-2, but not that of miR-133b (data not shown), suggesting that p38 signaling selectively regulates the transcription of miR-1/miR-133a clusters. In agreement of these results, we found MKK6E, a constitutively active activator of p38, was capable of increasing the miR-1/miR-133a enhancer activity (Figure 2D). In addition, we also measured the expression level of miR-1/miR-133 in p38α^f/f^ myoblasts infected with adenoviral Cre (Ad-Cre). As expected, we observed lower expression of miR-1/133 in myoblasts lacking p38α than that in control cells infected with adenoviral GFP (Ad-GFP) (Figure 2E). Moreover, we observed an increase in the expression level of miR-1/133 by adenoviral MKK6E infection, which provided further support for our hypothesis (Figure 2F). Taken together, these results indicate that p38 signaling is able to control the transcription of miR-1/miR-133a clusters.

### miR-133 Suppresses Cyclin D1 Expression and Induces G1 Phase Arrest in Myoblasts

**Figure 3:**
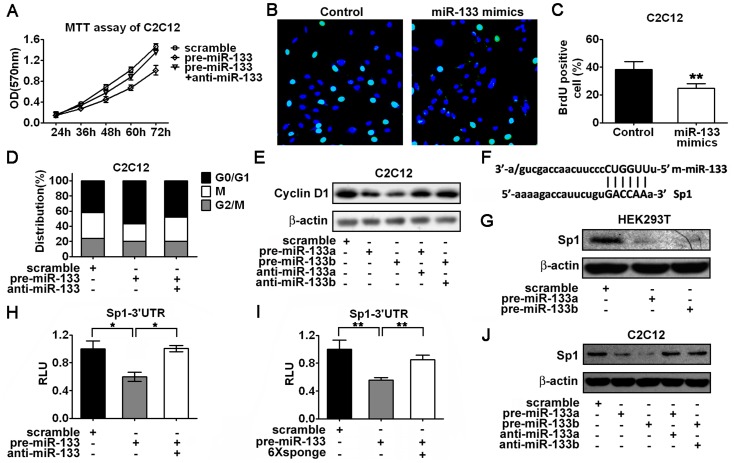
miR-133 induces G1 phase arrest and suppresses myoblast proliferation by downregualtion of Cyclin D1. (A) Growth curves of C2C12 myoblasts were determined by MTT assay. Cells were transfected with miR-133 precursors or anti-miR-133 as indicated. Error bars represent the standard deviation of three independent experiments. (B and C) Proliferation of C2C12 myoblasts was evaluated by BrdU staining. Cells were transfected with miR-133 mimics. Representative images of cells were taken by fluorescence microscope (B). The percentage of BrdU positive cells was measured (C). Data shown are from a typical experiment performed in triplicate. (D) Cell cycle analysis of C2C12 myoblasts transfected with miR-133 precursors or anti-miR-133 as indicated. Data shown are from a typical experiment performed. (E) Western blot analysis of Cyclin D1 protein expression in C2C12 myoblasts transfected with miR-133 precursors or anti-miR-133 as indicated. (F) Identification of miR-133 regulatory element in the 3′UTR of mouse Sp1. (G) Western blot analysis of Sp1 protein level in HEK293T cells transfected with miR-133 precursors as indicated. (H) Evaluation of miR-133 effect on a reporter containing Sp1–3× MRE in C2C12 myoblasts transfected with miR-133 precursors or anti-miR-133 as indicated. Error bars represent the SD of three independent experiments. (I) Determination of miR-133 effect on a reporter containing Sp1–3′UTR in C2C12 myoblasts transfected with miR-133 precursors or miR-133 specific sponges as indicated. Error bars represent the SD of three independent experiments. (J) Western blot analysis of Sp1 protein expression in C2C12 myoblasts transfected with miR-133 precursors or anti-miR-133 as indicated. *p<0.05, **p<0.01.

p38α is believed to be an antiproliferative factor in myoblast growth [22], [28], [29]. It has been shown that miR-1 and miR-133 also are involved in the regulation of proliferation. We hypothesized that miR-1 and miR-133 might mediate the antiproliferative effect of p38α signaling. To test this hypothesis, we first determined the effect of miR-133 on the cell growth of C2C12 myoblasts. Interestingly, we found that retroviral infection of miR-133a inhibited C2C12 myoblast proliferation (Figure S2A). Moreover, pooled miR-133 precursors, miR-133a mimics, and miR-133b mimics were all able to suppress C2C12 myoblasts growth (Figure 3A; Figure S2B). Additionally, inhibition of miR-133 by miR-133 antisense inhibitor (anti-miR-133) attenuated the suppressive effect of miR-133 on cell growth in C2C12 myoblasts (Figure 3A). Similar results were obtained in L6 myoblasts (Figure S2C). The antiproliferative effect of miR-133 on myoblast proliferation was then visualized and confirmed by BrdU staining in C2C12 and L6 myoblasts, respectively (Figure 3B; Figure S2D). Apparently, there were less BrdU positive cells after introducing miR-133 mimics into C2C12 or L6 myoblasts (Figure 3C; Figure S2E). Furthermore, G1 phase arrest induced by miR-133 precursor was observed in C2C12 myoblasts, which could be rescued by anti-miR-133 (Figure 3D). In accordance with these results, we found the mRNA and protein expression of Cyclin D1, a key regulator of G1/S phase transition, were all downregulated in the presence of miR-133 precursors, while inhibition of miR-133 by anti-miR-133 attenuated the repression (Figure 3E; Figure S2F).

### Sp1 is a Direct Target Gene of miR-133

However, according to the Targetscan software, Cyclin D1 is not a predicted target gene of miR-133. We then searched for transcriptional factors that might mediate the miR-133 effect on Cyclin D1 expression. According to the prediction by Targetscan, Sp1 is an evolutionarily conserved target of miR-133 (Figure 3F; Figure S3A). To determine whether Sp1 is a real target of miR-133, we first measured the mRNA and protein expression of Sp1 in HEK-293T cells transfected with miR-133 precursors. Our results show that miR-133 overexpression reduced the protein level of Sp1 but had no effect on the mRNA expression of Sp1, suggesting miR-133 functions at translational level (Figure 3G; Figure S3B). We next determined whether overexpression of miR-133 in myoblasts via retroviral infection would suppress the luciferase activity of the reporters containing Sp1–3× miRNA regulatory element (MRE) or Sp1–3′UTR as described in Materials and Methods. As expected, overexpression of miR-133a or miR-133b via an expression vector significantly repressed the luciferase activity of the reporters carrying either Sp1–3× MRE or Sp1–3′UTR (Figure S3C and S3D) in C2C12 myoblasts. Consistently, miR-133 precursors inhibited the luciferase activity of the reporter either containing Sp1–3× MRE or Sp1–3′UTR, while anti-miR-133 and miR-133 specific inhibitor (6× sponge) were able to attenuate the repression in C2C12 myoblasts (Figure 3H, I). The miR-133 mediated translational inhibition of Sp1 was further confirmed by western blot analysis in C2C12 and L6 myoblasts, respectively (Figure 3J; Figure S3E). In addition, anti-miR-133 rescued the repressive effect of miR-133 precursors on Sp1 protein level in C2C12 and L6 myoblasts, respectively (Figure 3J; Figure S3E). Taken together, our data indicate that Sp1 is a direct target gene of miR-133.

### Sp1 Regulates Cell Cycle Progression by Mediating Cyclin D1 Transcription in Myoblasts

**Figure 4:**
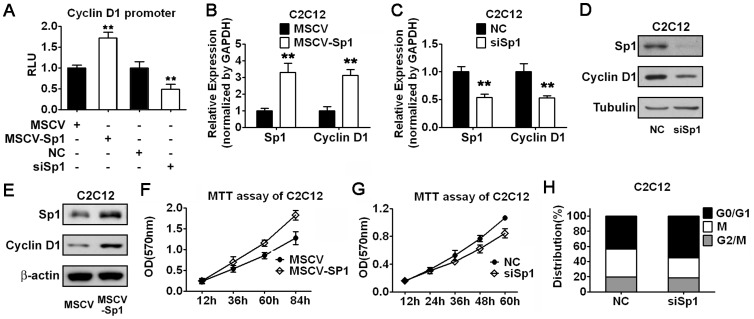
miR-133 downregulates Cyclin D1 expression via direct targeting Sp1. (A) Determination of Sp1 effect on the promoter activity of Cyclin D1. C2C12 myoblasts were cotransfected with a reporter containing Cyclin D1 promoter, and Sp1 expression vector (MSCV-Sp1), or control vector (MSCV), or Sp1 specific siRNA (siSp1), or negative control siRNA (NC) as indicated. Error bars represent the SD of three independent experiments. (B and C) Real-time RT-PCR analysis of Sp1 and Cyclin D1 mRNA expression in C2C12 myoblasts transfected with MSCV-Sp1 or MSCV (B), or transfected with siSp1 or NC (C), as indicated. Error bars represent the SD of three independent experiments. (D and E) Western blot analysis of Sp1 and Cyclin D1 protein level in C2C12 myoblasts transfected with siSp1 or NC (D), or MSCV-Sp1 or MSCV.(E). (F and G) Growth curves of C2C12 myoblasts were determined by MTT assay. Cells were transfected with MSCV-Sp1 or MSCV (F), or transfected with siSp1 or NC (G). Error bars represent the SD of three independent experiments. (H) Cell cycle analysis of C2C12 myoblasts transfected with siSp1 or NC as indicated. Data shown are from a typical experiment performed. **p<0.01.

Although there are predicted Sp1 sites in the promoter region of Cyclin D1, whether Sp1 could affect myoblast proliferation through the transcriptional regulation of Cyclin D1 has never been reported. To address this question, we performed luciferase assay using a reporter containing Cyclin D1 promoter. As shown in Figure 4A, in C2C12 myoblasts, overexpression of Sp1 increased the reporter activity, while knockdown Sp1 by specific siRNA decreased the reporter activity. In agreement of this result, increase of Sp1 expression resulted in upregulation of Cyclin D1 mRNA level, while knockdown of Sp1 led to downregulation of Cyclin D1 mRNA expression in C2C12 myoblasts (Figure 4B, C). Consistent with the real time RT-PCR results, Cyclin D1 protein levels were positively regulated by Sp1 as shown by western blot analysis (Figure 4D, E). These observations suggest that Cyclin D1 is a direct target gene of transcriptional factor Sp1 in myoblasts. To test whether Sp1-mediated Cyclin D1 expression contributes to myoblasts growth, we investigated the growth curve of C2C12 cells by MTT assay. As we expected, increased Sp1 expression promoted cell proliferation (Figure 4F), while reduced Sp1 expression suppressed cell growth (Figure 4G). Furthermore, we observed G1 phase arrest in C2C12 myoblasts transfected with Sp1 specific siRNA (Figure 4H), which recaptured the effect of miR-133. Consistent with our hypothesis, miR-133 only altered the mRNA expression of Cyclin D1 but did not affect the Sp1 mRNA expression in C2C12 myoblasts (Figure S2F).

### miR-1 Induces G1 Phase Arrest by Direct Targeting Cyclin D1 Expression in Myoblasts

**Figure 5:**
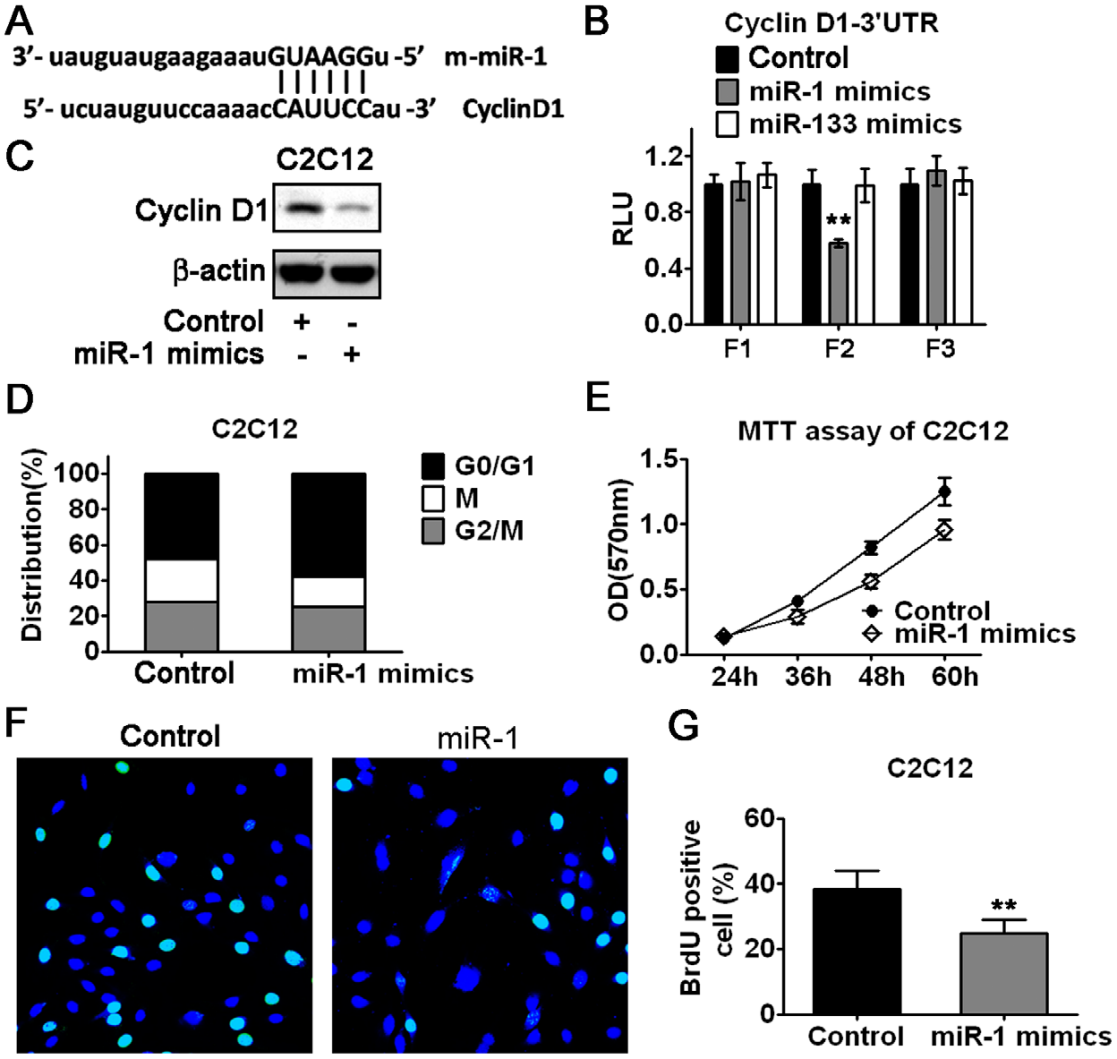
miR-1 induces G1 phase arrest and inhibits myoblast proliferation by direct targeting Cyclin D1. (A) Identification of miR-1 regulatory element in the 3′UTR of mouse Cyclin D1. (B) Evaluation of effect of miR-1 mimics and miR-133 mimics on reporters containing three different regions of Cyclin D1–3′UTR in C2C12 myoblasts transfected with miR-1 or miR-133 mimics as indicated. Error bars represent the SD of three independent experiments. (C) Western blot analysis of Cyclin D1 protein expression in C2C12 myoblasts transfected with miR-1 mimics or control oligos. (D) Cell cycle analysis of C2C12 myoblasts transfected with miR-1 mimics or control oligos. Data shown are from a typical experiment performed. (E) Growth curves of C2C12 myoblasts were determined by MTT assay. Cells were transfected with miR-1 mimics or control oligos. Error bars represent the SD of three independent experiments. (F and G) Proliferation of C2C12 myoblasts was evaluated by BrdU incorporation. Cells were transfected with miR-1 mimics. Representative images of cells were taken by fluorescence microscope (F). The percentage of BrdU positive cells was measured (G). Data shown are from a typical experiment performed in triplicate. **p<0.01.

miR-1 has been reported to inhibit myoblast proliferation, however, the molecular mechanisms still need to be delineated. When we searched for miR-133 regulatory element in the 3′UTR of Cyclin D1, we happened to notice that Cyclin D1 is a predicted target of miR-1 (Figure 5A; Figure S4A). We next determined whether overexpression of miR-1 would suppress the luciferase activity of the reporters containing three different regions of Cyclin D1–3′UTR (Figure S4B) as described in Materials and Methods. Consistent with the prediction by Targetscan, miR-1 selectively repressed the luciferase activity of the reporter carrying miR-1 regulatory element (F2), while miR-133 had no effect on the luciferase activity of all the reporters tested here (Figure 5B). To test whether miR-1 could inhibit Cyclin D1 protein expression, we performed western blot analysis. As shown in Figure 5C, introduction of miR-1 mimics into C2C12 myoblasts resulted in the attenuation of Cyclin D1 protein expression. Since Cyclin D1 is a key regulator for G1/S phase transition, we were not surprised to observe G1 phase arrest in C2C12 myoblasts transfected with miR-1 mimics (Figure 5D). As a consequence of G1 phase arrest, the proliferation of C2C12 myoblasts was inhibited by miR-1 mimics (Figure 5E). The suppressive effect of miR-1 on the proliferation of C2C12 myoblasts was further confirmed by BrdU staining (Figure 5F, G). Similar results were obtained in L6 myoblasts (Figure S4C, S4D, and S4E). Our results suggest that miR-1 is able to repress myoblast proliferation through targeting Cyclin D1-mediated G1/S transition.

### Downregulation of p38 Signaling Leads to Upregulation of Cyclin D1 in Myoblasts

**Figure 6:**
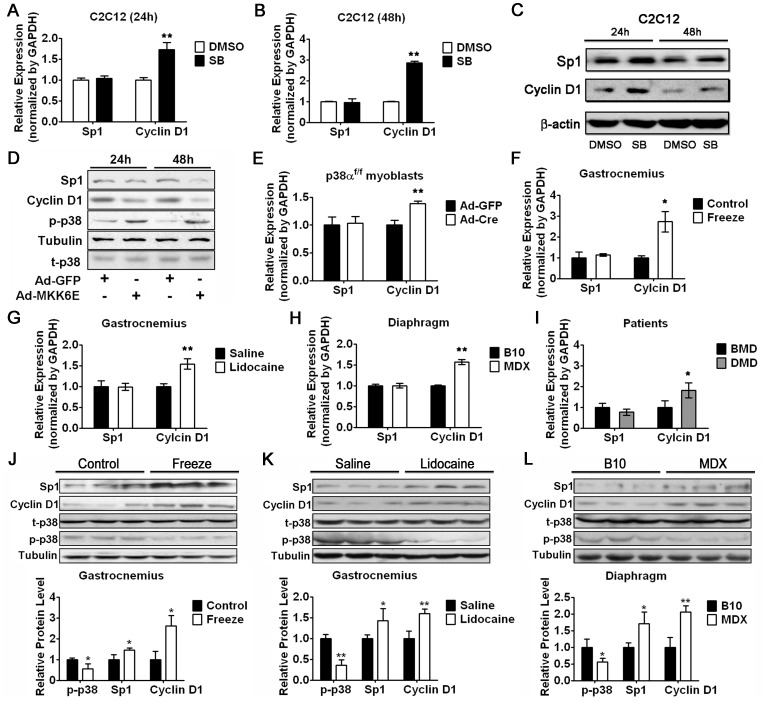
Sp1 and Cylin D1 expression are downregulated during Early Phase of Muscle Regeneration. (A and B) Real-time RT-PCR analysis of Sp1 and Cyclin D1 mRNA expression in C2C12 myoblasts treated with SB203580 or DMSO as a control for 24 hours (A) or 48 hours (B) as indicated. Error bars represent the SD of three independent experiments. (C) Western blot analysis of Sp1 and Cyclin D1 protein expression in C2C12 myoblasts treated with SB203580 or DMSO as a control for 24 hours or 48 hours as indicated. (D) Western blot analysis of Sp1, Cyclin D1 and p-p38 protein level in C2C12 myoblasts infected with Ad-MKK6E or Ad-GFP as a control for 24 hours or 48 hours as indicated. (E) Real-time RT-PCR analysis of Sp1 and Cyclin D1 mRNA expression in p38α^f/f^ myoblasts infected with Ad-Cre or Ad-GFP as a control as indicated. Error bars represent the SD of three independent experiments. (F–I) Real-time RT-PCR analysis of the expression of Sp1 and Cyclin D1 in GAS muscle of mice following freeze injury (F), in GAS muscle of mice following lidocaine injection (G), in Diaphragm muscle of mdx mice (H), in skeletal muscle of patients with BMD or DMD (I). SDs are shown as error bars (n≥3). (J–L) Western blot analysis of Sp1, Cyclin D1, and p-p38 in muscle regeneration mouse models, including freeze injury model (J), lidocaine injury model (K), and mdx mouse model (L). The relative intensity of each band was quantified with NIH image. Tubulin and t-p38 were used for normalization for Sp1/Cyclin D1, and p-p38 respectively. SDs are shown as error bars (n = 3). *p<0.05, **p<0.01.

Based on our results, we hypothesized that downregulation of p38 signaling, which would decrease the expression level of miR-1/133, would lead to derepression of Cyclin D1. In deed, we found that the mRNA expression of Cyclin D1 increased in a time-dependent manner in C2C12 myoblasts treated with SB203580 (Figure 6A, B). In agreement of the finding that Sp1 is regulated by miR-133 at posttranscriptional level, we did not detect any change in the mRNA level of Sp1 (Figure 6A, B). Consistently, the protein expression of Sp1 and Cyclin D1 was elevated by SB203580 treatment (Figure 6C). In contrast, the protein expression of Sp1 and Cyclin D1 was downregulated in myoblasts infected with Ad-MKK6E (Figure 6D). Since our data in Figure 2E show that miR-1/133 expression was lower in myoblasts lacking p38α, we speculated that the mRNA expression of Cyclin D1 should be upregulated in these cells. As expected, only Cyclin D1 but not Sp1 mRNA level was elevated in p38α^f/f^ myoblasts infected with Ad-Cre (Figure 6E). These findings further support the idea that downregulation of p38-mediated miR-1/133a promotes myoblast proliferation by activating Cyclin D1 expression. Taken together, our *in vitro* experiments reveal a novel mechanism by which p38-mediated muscle specific miR-133 and miR-1 affect myoblast cell cycle progression via their direct target genes, Sp1 and Cyclin D1.

### p38 Activity Decreases and Sp1/Cyclin D1 Expression Increases during Early Muscle Regeneration

Based on our *in vitro* data, we hypothesized that p38 activity would be low in regenerating muscles and the expression of miR-1/133 and their targets Sp1 and Cyclin D1 would be reversely correlated during muscle regeneration. To test our hypothesis, we first investigated the mRNA levels of Sp1 and Cyclin D1 in the muscle of mice received freeze treatment or lidocaine treatment. Consistent with our observation *in vitro*, since miR-133 acts at translational level, the mRNA expression of Sp1 did not alter, while the Cyclin D1 transcripts significantly increased after either treatment (Figure 6F, G), indicating active proliferation of myogenic satellite cells after muscle damage. In addition, we also evaluated the mRNA expression of Sp1 and Cyclin D1 expression in mdx mice and patients with mild Becker muscular dystrophy (BMD) and severe Duchenne muscle dystrophy (DMD). As shown in Figure 6H, Cyclin D1 but not Sp1 transcripts were increased in regenerating muscles derived from mdx mice. As expected, Cyclin D1 was higher in skeletal muscle of patients with severe DMD as compared to patients with mild BMD (Figure 6I). The protein levels of Sp1 and Cyclin D1 were also determined in mouse GAS muscle following freeze injury or lidocaine injection, as well as in mdx mice. As we expected, the protein levels of Sp1 and Cyclin D1 were all elevated in regenerating muscles (Figure 6J–L). These results together with the finding in Figure 1 suggest that the expression level of miR-1/133 is reversely correlated with their targets Cyclin D1 and Sp1 in regenerating muscles. In agreement of our hypothesis that p38 signaling is one of the upstream signalings of miR-1/133, we observed downregulation of p38 activity in regenerating muscle of mice received freeze or lidocaine treatment, as well as in mdx mice muscles (Figure 6J–L).

### Proproliferative FGF2 Inhibits p38 Signaling

**Figure 7:**
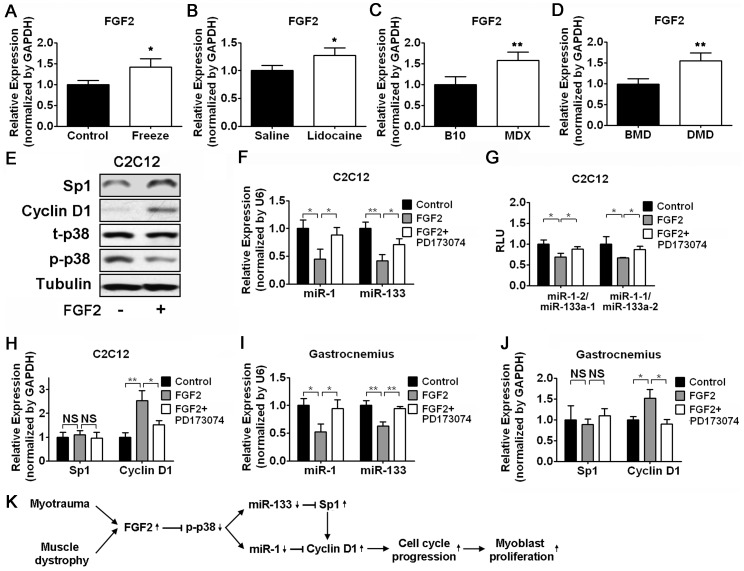
Proproliferative FGF2 attenuates p38 activity and miR-1/133 expression in myoblasts. (A–D) Evaluation of FGF2 mRNA expression by real-time RT-PCR in regenerating muscle following freeze injury (A), or following lidocaine injection (B), or in the muscle of mdx mice (C) or patients with BMD or DMD (D). SDs are shown as error bars (n≥3). (E) Western blot analysis of p-p38, Sp1 and Cyclin D1 in C2C12 myoblasts treated with FGF2 or vehicle. (F) Real-time RT-PCR analysis of miR-1/133 expression in C2C12 myoblasts treated with FGF2 and FGF2 signaling inhibitor (PD173074), as indicated. Error bars represent the SD of three independent experiments. (G) Determination of the effect of FGF2 and PD173074 on enhancer activity of miR-1–2/miR-133a-1 or miR-1–1/miR-133a-2 cluster in C2C12 myoblasts, as indicated. Error bars represent the SD of three independent experiments. (H) Real-time RT-PCR analysis of Sp1 and Cyclin D1 mRNA expression in C2C12 myoblasts treated with FGF2 and PD173074 as indicated. Error bars represent the SD of three independent experiments. (I and J) Real-time RT-PCR analysis of miR-1/133 (I), Sp1 (J), and Cyclin D1 (J) expression in isolated GAS muscle treated with FGF2 and PD173074 as indicated. Error bars represent the SD of three independent experiments. (K) Schematic representation of a novel mechanism of FGF2 stimulated myoblast proliferation during muscle regeneration. FGF2-induced attenuation of p38-mediated miR-1/133 expression facilities myoblasts proliferation via derepressing miR-1/133 direct downstream targets Sp1/Cyclin D1. *p<0.05, **p<0.01.

In a search for potential causes for the downregulation of p38 signaling during muscle regeneration, we wondered whether growth factors could be involved. As previous reported, the release of proproliferative FGF2 in myotrauma is proportional to the degree of injury and there is a positive correlation between the level of FGF2 and the speed of muscle regeneration. In agreement of this concept, we found the mRNA level of FGF2 was upregulated in the mouse skeletal muscle following freeze injury or lidocaine injection (Figure 7A, B). Similarly, the mRNA expression of FGF2 was increased in the skeletal muscle from mdx mice (Figure 7C). Moreover, as compared to patients with mild BMD, patients with severe DMD have higher FGF2 levels (Figure 7D). To test whether FGF2 could repress p38 activity and whether p38-mediated miR-1/133 transcription was affected by FGF2, we determined the p38 activity and the expression level of miR-1/133 in C2C12 myoblasts treated with FGF2. As expected, FGF2 treatment resulted in reduced p38 activity and decreased level of miR-1/133 (Figure 7E, F). In addition, the enhancer activity of miR-1/133a clusters was inhibited by FGF2 (Figure 7G). Consistent with the concept that FGF2 promotes myoblasts proliferation, increased protein level of Sp1 and Cyclin D1 was observed after FGF2 treatment (Figure 7E). In contrast, only the mRNA expression of Cyclin D1 but not Sp1 was upregulated by FGF2 addition (Figure 7H). Furthermore, the downregulation of miR-1/133 expression and upregulation of Cyclin D1 mRNA expression by FGF2 were observed in isolated GAS muscles (Figure 7I, J). When FGFR1 specific inhibitor PD173074 was used to block FGF2 signaling, the inhibitory effect of FGF2 on the transcription of miR1/133 was attenuated (Figure 7F, G, I). As we expected, the induction of Cyclin D1 expression by FGF2 was blocked by PD173074 treatment (Figure 7H, J). Our *in vivo*, *in vitro*, and *ex vivo* data indicate that FGF2 might promote myogenic satellite cell proliferation by stimulating cell cycle progression through a p38/miR-1/133-mediated mechanism.

Taken together, our results suggest a novel mechanism of FGF2 stimulated myoblast proliferation during muscle regeneration, which involves attenuation of p38-mediated miR-1/miR-133 transcription and upregulation of their direct downstream targets Sp1 and Cyclin D1 (Figure 7K).

## Discussion

Understanding the molecular basis underlying muscle regeneration is crucial for better management of muscle dystrophy and muscle injury. Growing evidence has demonstrated that muscle specific miRNAs functions as a control center in directing diverse biological processes during myogenic proliferation and differentiation. Modulation of the expression of various miRNAs at different stages during muscle regeneration may provide novel strategies to manipulate either proliferation or differentiation. Here we are attempting to focus our study on myoblast proliferation, an early stage of the muscle regeneration. Our results unravel a novel function of miR-1 and miR-133 in regulating cell cycle progression of myoblasts via direct and indirect targeting Cyclin D1, a key regulator of G1/S phase transition. The downregulation of these two antiproliferative miRNAs during early stage of muscle regeneration could facilitate myoblast proliferation, which results in accumulation of more myoblasts ready for differentiation.

miRNAs exhibit enormous regulatory potential and are able to target multiple genes in one signaling pathway or one regulatory network, which in turn is targeted by multiple miRNAs in combination fashion. Here we demonstrated that miR-1/133a clusters were transcribed in response to one signaling (p38 signaling), then targeted two key components (Sp1 and Cyclin D1) in the cell cycle regulatory network, and worked in concert to coordinate one biological process. Our studies together with other reports suggest the existence of a complex network composed of multiple miRNAs and a host of cell cycle regulators that control the myoblast proliferation. All the components work together efficiently to achieve a common goal in response to external stimuli.

There is an earlier paper that shows miR-133 promotes skeletal myoblast proliferation and inhibits differentiation [8]. While another two papers show miR-133 represses cardiac muscle proliferation [10] and inhibits cardiac hypertrophy [11]. It remains controversial whether miR-133 promotes or inhibits muscle cell proliferation. The current manuscript shows that miR-133 inhibit skeletal myoblast proliferation. This discrepancy might be due to different experimental conditions. The role of miR-133 in skeletal muscle regarding proliferation remains to be further studied.

The formation of skeletal muscle is a well-orchestrated multistep process. Proliferation and differentiation are mutually exclusive processes in myogenesis. Previous studies provide evidence that p38α not only promotes muscle differentiation and fusion, but it is a critical regulator of myoblast cell cycle exit, a necessary step prior to commencing the muscle differentiation gene program. Our studies indicate that the attenuation of p38 signaling at the early stage of muscle regeneration might be essential for myoblast proliferation. Our data together with others suggest that p38 activation needs to be tightly controlled at different stages throughout the lifespan of muscle formation. In addition, we provided first evidence that p38 controls myoblast proliferation via a miRNA-mediated mechanism.

The process of muscle regeneration requires FGFs and a sequence of cellular events, which results in the regulation of the satellite cell population. Although the instructive role of the FGFs is well documented, the intracellular signaling that converts the proliferative cues released in the regenerative environment into the epigenetic information that coordinates myoblast proliferation is unknown. It has been reported that FGF signaling through ERK MAP kinase delays the expression of miR-1 and miR-133 in cells undergoing myogenic differentiation [30]. However, whether FGF have effects on the expression of miR-1 and miR-133 in proliferating myoblasts as well as in regenerating muscles, and which signaling pathway transduces the FGF signaling are not known. Here we propose that FGF2 released from the myotrauma represses the p38 signaling and expression of miR-1/133, and the repressed p38 signaling and subsequent downregulation of miR-1/133 in turn facilitate the satellite cell proliferation at the early stages of muscle regeneration.

Taken together, our studies reveal essential roles of miR-1/133 in coordinating myoblast proliferation and point to miR-1/133 as critical components of FGF2/p38 regulatory circuit that regulates myoblast expansion during the early stages of muscle regeneration (Figure 7K). Nevertheless, many details of miR-1/133 regulation of myogenic proliferation and differentiation remain to be elucidated. The identification of new targets for miR-1/133 as well as the upstream signaling mechanisms and extracellular activating cues will undoubtedly increase our understanding of how miR-1/133 regulates muscle regeneration.

## Materials and Methods

### Ethics Statement

Our study was reviewed and approved by the Institutional Review Board of Institute for Nutritional Sciences, Shanghai Institutes for Biological Sciences, Chinese Academy of Sciences, and Ethics Committee of Pediatrics Clinical Pharmacology, Fudan University. Animals were maintained and experiments were performed according to protocols approved by the Animal Care and Use Committees of Institute for Nutritional Sciences (permit number: 2011-AN-14). BMD and DMD patient specimens were obtained surgically from patients after obtaining their written informed consent.

### Plasmid and RNA Oligonucleotide

miRNA-expressing vectors, pSIF-133a and pSIF-133b, were gifts from Dr. Yong Li (University of Louisville, US). pcDNA-MKK6E and pAd-Cre plasmids were gifts from Dr. Lijian Hui (SIBS, CAS, China). MKK6E cDNA was amplified by PCR using pcDNA-MKK6E as a template, and the PCR product was subcloned into retroviral vector MSCV-IRES-GFP. Mouse Sp1 cDNA amplified by RT-PCR was subcloned into the XhoI and EcoR sites of MSCV vector to obtain MSCV-Sp1. The miR-133a1 precursor was amplified by PCR using mouse genomic DNA as a template, and the PCR product was cloned into MDH1-IRES-GFP vector (Addgene) to generate miRNA expression plasmids. The primer sequences used for cloning are provided in Table S1.

For construction of the luciferase reporter plasmid, the 3′UTR of Sp1 or Cyclin D1 was amplified from mouse cDNA by PCR, and inserted into pRL-TK vector. The construction for reporter pRL-3× MRE (miRNA regulatory element), which contains three repeats of MRE), was mentioned previously [31]. Oligonucleotide sequences are provided in Table S1. Enhancer or possible promoter sequences of miR-1–2/miR-133a1, miR-1–1/miR-133a2, and miR-133b containing E-box and MEF2 sites, were amplified by using mouse genomic DNA as a template and cloned into pTK109-luc vector.

Sponges designed as decoy targets for microRNAs were effective and specific inhibitors of microRNA seed families. The method of making miRNA sponge plasmid was described previously [32]. In brief, oligonucleotides of miR-133a binding site (CAGCTGGTTGTCCGGACCAAA) with 10 bp linker sequences containing XhoI and EcoRI sites were chemically synthesized. To obtain MDH1–6× miR-133a sponge plasmid with six tandemly arrayed miRNA-133a binding sites, the oligonucleotides were annealed, ligated, and inserted into MDH1-IRES-GFP vector.

Pre-miR™ miRNA Precursor and Anti-miR™ miRNA Inhibitor of miR-133a or miR-133b were purchased from Ambion. GMR-miR™ microRNA double-stranded mimics for miR-133a or miR-133b were obtained from Genepharma. The control siRNA and siSp1 RNA oligos were purchased from Genepharma. The siSp1 sequences were as follows: sense (5′–3′) UUGAGUCACCCAAUGAGAAtt, antisense (5′–3′) UUCUCAUUGGGUGACUCAAtt.

### Real-time RT-PCR and Western Blot Analysis

Total RNA was isolated by using Trizol reagent (Invitrogen) according to the manufacturer’s instructions. Small RNA (≤200 nucleotides) was extracted from tissue and cells by using mirVana™ miRNA Isolation kit (Ambion) according to the manufacturer’s instructions. Total RNA was reverse-transcribed by using PrimeScript RT reagent Kit (TaKaRa). Small RNA polyadenylation were performed according to the protocols described before [33]. Real-time PCR was performed on an ABI 7900 Real-Time PCR System (Applied Biosystems). The primer sequences are provided in Table S2. Western Blot analysis was performed as described before with minor modification [34]. Briefly, cultured cells and mouse tissues were homogenized in RIPA lysis buffer (50 mM Tris-HCl, pH 7.5, 150 mM NaCl, 1.0 mM EDTA, 0.1% SDS, 1% Sodium deoxycholate, and 1% Triton X-100). The buffer was supplemented with Protease Inhibitor Cocktail and Phosphatase Inhibitor Cocktail (Sigma) just prior to use. The protein concentration was determined using a BCA protein assay kit (Thermo Fisher). Protein lysates were resolved on 10% SDS-PAGE gels using standard procedures. Anti-Sp1 (Santa Cruz), anti-Cyclin D1 (Santa Cruz), anti-α-tubulin (Sigma), anti–β-actin (Sigma), and anti-phospho-p38 (Cell Signaling) antibodies were used for western blot analysis.

### Cell Culture, Transfection and Luciferase Assay

Mouse C2C12 myoblasts (ATCC) and rat L6 myoblasts (Dr. Jia Li, SIBS, CAS, China) [35] were maintained in a humidified incubator at 37°C and 5% CO_2_ in Dulbecco’s modified Eagle medium (DMEM) containing 10% fetal bovine serum, and 1% penicillin and streptomycin (Gibco). Transfection was performed using Lipofectamine 2000 (Life Tech.) according to the standard protocol. The p38α and p38β inhibitor, SB203580 (final concentration 10 µM), was purchased from Sigma. Fibroblast Growth Factor-Basic Human Recombinant (FGF2) was from ProSpec (final concentration 10 ng/ml). FGFR1 inhibitor PD173074 was from Han-Xiang Chemical (final concentration 10 nM). Luciferase assays were performed by using the Dual-Luciferase Reporter Assay System (Promega) following the manufacturer’s protocol. Cells were harvested 48 hours after transfection. Luciferase activities were measured on a luminometer (Berthold Technologies).

### Proliferation Analysis and Cell Cycle Analysis

MTT assay: Cells were seeded into 96-well plates and grown for 24 hours after transfection. At the time indicated, cells were incubated with 0.5 mg/ml 3-(4, 5-dimethylthiazol–2-yl)-2, 5-diphenyltetrazolium bromide (MTT, Sigma) for 4 hours at 37°C. The media was carefully removed from each well and 150 µl of DMSO was added. The plates were then measured at 570 nm using a multiwell spectrophotometer (Molecular Devices, Inc.).

BrdU incorporation assay: Cells were incubated with 10 µg/mL BrdU (Sigma) for 2 hours before fixed with ice cold methanol, and then rinsed twice with PBS. The fixed cells were further treated with 2 N HCl/1% Triton X-100 for 30 minutes. After three rinses with PBS, 0.1 M sodium borate was added for 2 minutes. Next, the cells were incubated with anti-BrdU antibody (1∶50, Zhongshan Goldbridge Biotechnology) overnight at 4°C. Alexa Fluor 488 goat anti-mouse IgG1 (1∶1000, Invitrogen) was added as a secondary antibody, and the cells were incubated for 1 hour at room temperature. Finally, all cells were rinsed three times with PBS; cell nuclei were counterstained with DAPI Solution (Thermo Fisher) for 5 minutes. And then images were taken with a fluorescent microscope (Olympus IX71), and the mean ± SD of cells with BrdU incorporation was calculated.

Cell cycle analysis was performed 48 hours after transfection. Cell cycle phase distribution was analyzed by flow cytometry. Briefly, Cells were trypsinized, washed with PBS, then fixed in 1 ml 70% ethanol, and stored at 4°C overnight before DNA analysis. After the removal of ethanol by centrifugation, cells were incubated with 0.5% Triton X-100, RNase A (100 mg/ml) and stained with propidium iodide (50 mg/ml, Sigma) for 60 minutes in the dark. The DNA content was analyzed by a Cell Lab Quanta SC flow cytometer (Beckman Coulter). Resulting DNA distributions were analyzed by Modfit (Verity Software House) to determine the proportions of cells in the various phases of the cell cycle.

### Animals and Patient Specimens

p38α^f/f^ mice were gifts from Dr Lijian Hui (SIBS, CAS, China) [36]. Several animal models of muscle regeneration were used in the study. Freeze injury model was induced by a single freeze injury of the GAS muscle as described before subject to slightly modification [37]. Under anesthesia, the skin of the GAS muscle were shaved and exposed. Injury was induced by applying a steel bar cooled in liquid nitrogen to the GAS muscle for 20s. Lidocaine is reported to cause rapid destruction of muscle fibers via nerve block [38]. Lidocaine injury model was induced by a single dose intramuscular injection of 200 µl 2% lidocaine into the right GAS muscle. The left GAS muscle of the same animal received 0.9% saline injection and served as a control group. Seven days after injection or at indicated time points, the GAS muscles were isolated and examined.

### Retroviral and Adenoviral Preparation

To produce retrovirus, standard procedures were performed as described before [39]. Briefly, supernatant contained high-titer retrovirus was generated by co-transfection of a retrovirus vector MDH1/MSCV and the pCL-eco viral packaging construct into HEK-293T cells (ATCC). ViraPower™ Adenoviral Expression System (Invitrogen) was used in this study according to the user manual.

### Primary Myoblasts Isolation

The protocol used was described previously [40]. In brief, trimmed and minced muscle tissue was incubated with 0.1% Pronase in DMEM at 37°C on a rocker for 1 hour. After removal of supernatant by centrifugation, the pellet was resuspended and filtered with a 40 µm nylon mesh cell strainer (Falcon 2350). To enrich the myoblasts, cells were collected by centrifugation, resuspended and preplated in a noncoated dish for 1 hour to remove fibroblasts. The floating cells with enriched myoblasts were then transferred to Matrigel-coated plates (BD Biosciences).

### Statistical Analysis

For All Data are expressed as means ± standard deviation (SD) and analyzed by unpaired Student’s t-test for statistical significance. p values of <0.05 were considered to be significant.

## Supporting Information

Figure S1 **miR-1/133 expression are decreased in regenerating muscle tissues.** (A) Hematoxylin and eosin staining of GAS muscle of mice at 7 days following freeze injury or lidocaine injection, as indicated. (B) Real-time RT-PCR analysis of the expression of miR-1/133 in GAS muscle of mice following freeze injury or lidocaine injection. (C) Real-time RT-PCR analysis of the time-course expression of regeneration markers, TNFα and IL-6, in GAS muscle of mice following freeze injury. Data shown are from a typical experiment performed. SDs are shown as error bars (n≥3), **p<0.01.
(TIF)

Figure S2 **miR-133 suppresses myoblast proliferation.** (A–C) Growth curves of C2C12 myoblasts were determined by MTT assay. Cells were transfected with miR-133a expression vector (MDH1-miR-133a) (A), or miR-133 mimics (B), or miR-133 precursors or anti-miR-133 (C), as indicated. Error bars represent the SD of three independent experiments. (D and E) Proliferation of L6 myoblasts was evaluated by BrdU incorporation. Cells were transfected with miR-133 mimics. Representative images of cells were taken by fluorescence microscope (D). The percentage of BrdU positive cells was measured (E). Data shown are from a typical experiment performed in triplicate. (F) Real-time RT-PCR analysis of Sp1 and Cyclin D1 mRNA expression in C2C12 myoblasts transfected with miR-133 precursors. Error bars represent the SD of three independent experiments. **p<0.01.
(TIF)

Figure S3 **Sp1 is a target gene of miR-133.** (A) Comparison of miR-133 regulatory elements in the 3′UTR of Sp1 across species. (B) Real-time RT-PCR analysis of Sp1 mRNA expression in HEK293T cells transfected with miR-133 precursors. Error bars represent the SD of three independent experiments. (C and D) Determination of miR-133 effect on reporters containing Sp1–3× MRE (C) or Sp1–3′UTR (D) in C2C12 myoblasts transfected with miR-133 expression vector (pSIF-133) or empty vector (pSIF) as indicated. Error bars represent the SD of three independent experiments. (E) Western blot analysis of Sp1 protein expression in L6 myoblasts transfected with miR-133 precursors or anti-miR-133 as indicated. *p<0.05, **p<0.01.
(TIF)

Figure S4 **miR-1 inhibits myoblast proliferation via targeting Cyclin D1.** (A) Comparison of miR-1 regulatory elements in the 3′UTR of Cyclin D1 across species. (B) Schematic representation of reporters containing three different regions of Cyclin D1–3′UTR, designated F1, F2 and F3. (C) Growth curves of L6 myoblasts were determined by MTT assay. Cells were transfected with miR-1 mimics or control oligos. Error bars represent the SD of three independent experiments. (D and E) Proliferation of L6 myoblasts was evaluated by BrdU incorporation. Cells were transfected with miR-1 mimics. Representative images of cells were taken by fluorescence microscope (D). The percentage of BrdU positive cells was measured (E). Data shown are from a typical experiment performed in triplicate. **p<0.01.
(TIF)

Table S1 **Oligos used in cloning.**
(DOC)

Table S2 **Primers used in qRT-PCR.**
(DOC)
