# Supplementary figures and images for: Attenuation of p38-Mediated miR-1/133 Expression Facilitates Myoblast Proliferation during the Early Stage of Muscle Regeneration
Source: PLoS One. 2012 Jul 24;7(7):e41478. doi: 10.1371/journal.pone.0041478 (PMC3404058; doi:10.1371/journal.pone.0041478)

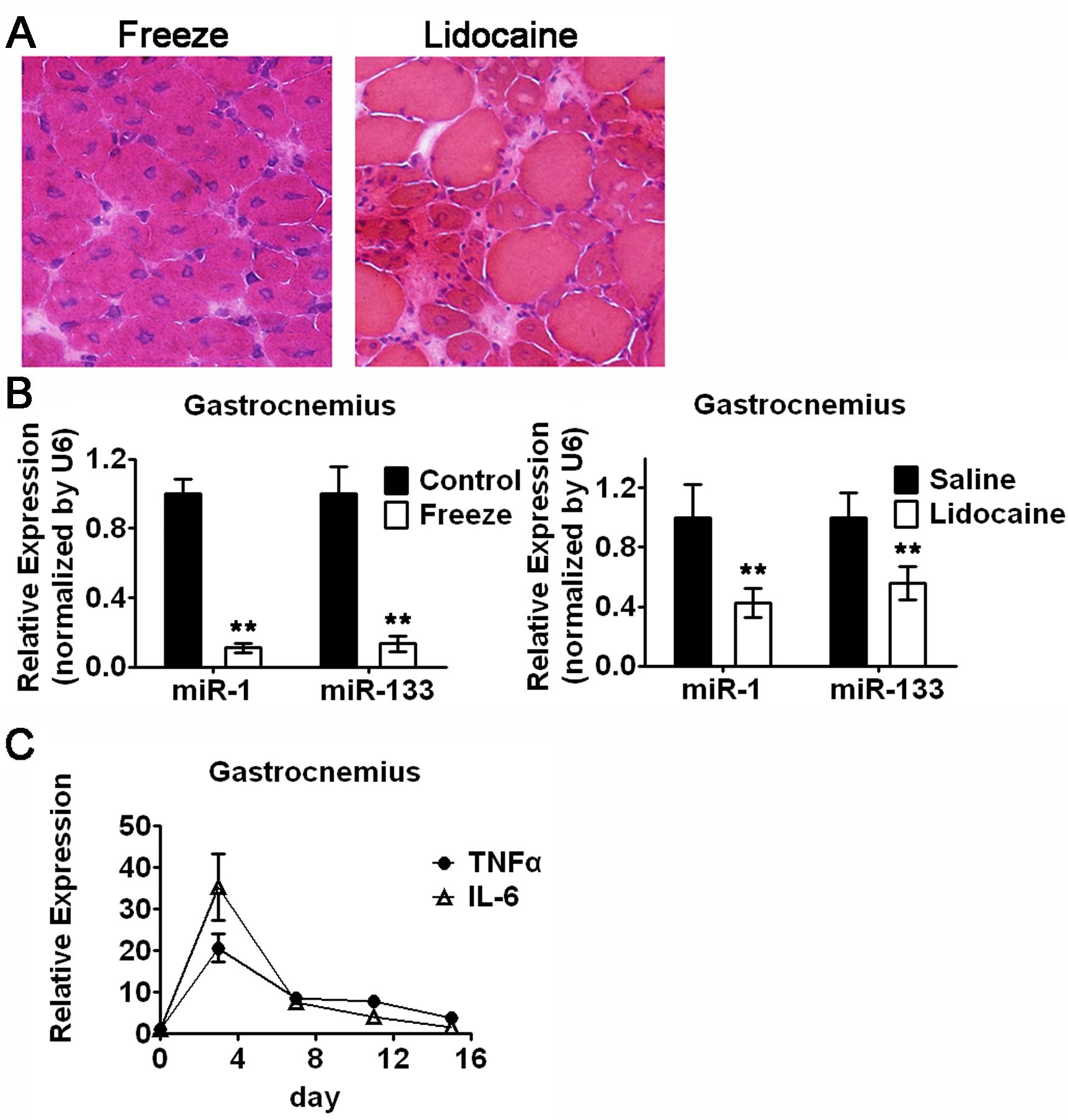

Supplement: Figure S1 — miR-1/133 expression are decreased in regenerating muscle tissues. (A) Hematoxylin and eosin staining of GAS muscle of mice at 7 days following freeze injury or lidocaine injection, as indicated. (B) Real-time RT-PCR analysis of the expression of miR-1/133 in GAS muscle of mice following freeze injury or lidocaine injection. (C) Real-time RT-PCR analysis of the time-course expression of regeneration markers, TNFα and IL-6, in GAS muscle of mice following freeze injury. Data shown are from a typical experiment performed. SDs are shown as error bars (n≥3), **p<0.01. (TIF) [file pone.0041478.s001.tif]

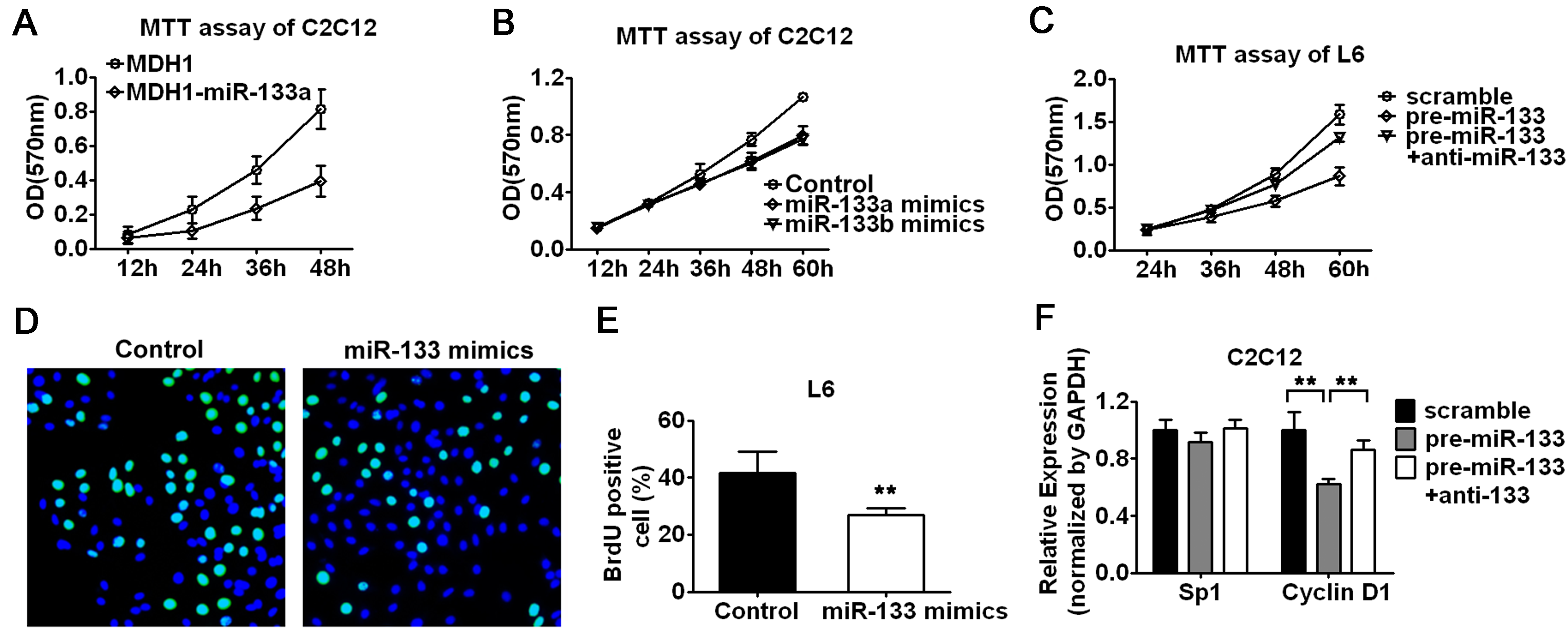

Supplement: Figure S2 — miR-133 suppresses myoblast proliferation. (A–C) Growth curves of C2C12 myoblasts were determined by MTT assay. Cells were transfected with miR-133a expression vector (MDH1-miR-133a) (A), or miR-133 mimics (B), or miR-133 precursors or anti-miR-133 (C), as indicated. Error bars represent the SD of three independent experiments. (D and E) Proliferation of L6 myoblasts was evaluated by BrdU incorporation. Cells were transfected with miR-133 mimics. Representative images of cells were taken by fluorescence microscope (D). The percentage of BrdU positive cells was measured (E). Data shown are from a typical experiment performed in triplicate. (F) Real-time RT-PCR analysis of Sp1 and Cyclin D1 mRNA expression in C2C12 myoblasts transfected with miR-133 precursors. Error bars represent the SD of three independent experiments. **p<0.01. (TIF) [file pone.0041478.s002.tif]

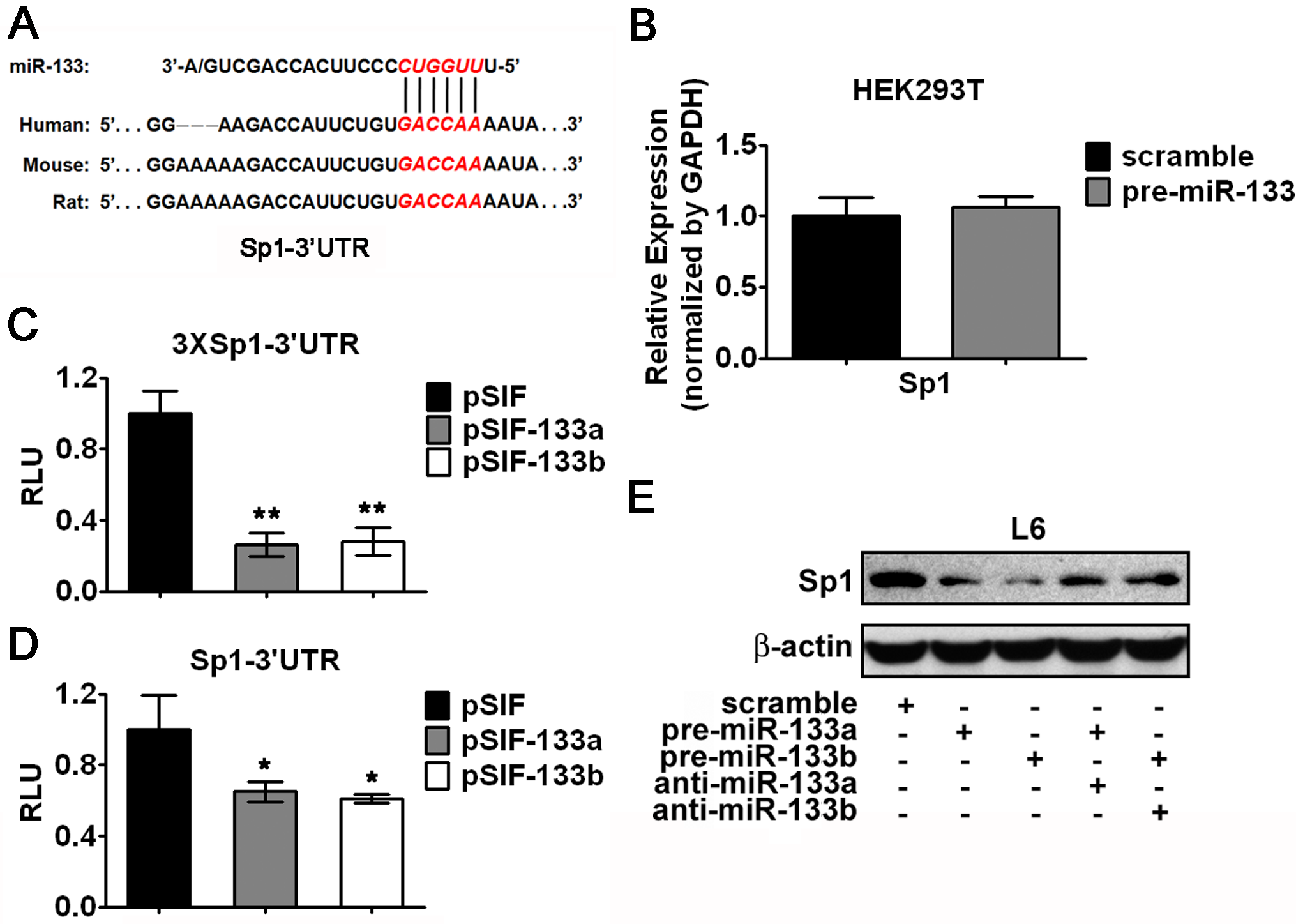

Supplement: Figure S3 — Sp1 is a target gene of miR-133. (A) Comparison of miR-133 regulatory elements in the 3′UTR of Sp1 across species. (B) Real-time RT-PCR analysis of Sp1 mRNA expression in HEK293T cells transfected with miR-133 precursors. Error bars represent the SD of three independent experiments. (C and D) Determination of miR-133 effect on reporters containing Sp1–3× MRE (C) or Sp1–3′UTR (D) in C2C12 myoblasts transfected with miR-133 expression vector (pSIF-133) or empty vector (pSIF) as indicated. Error bars represent the SD of three independent experiments. (E) Western blot analysis of Sp1 protein expression in L6 myoblasts transfected with miR-133 precursors or anti-miR-133 as indicated. *p<0.05, **p<0.01. (TIF) [file pone.0041478.s003.tif]

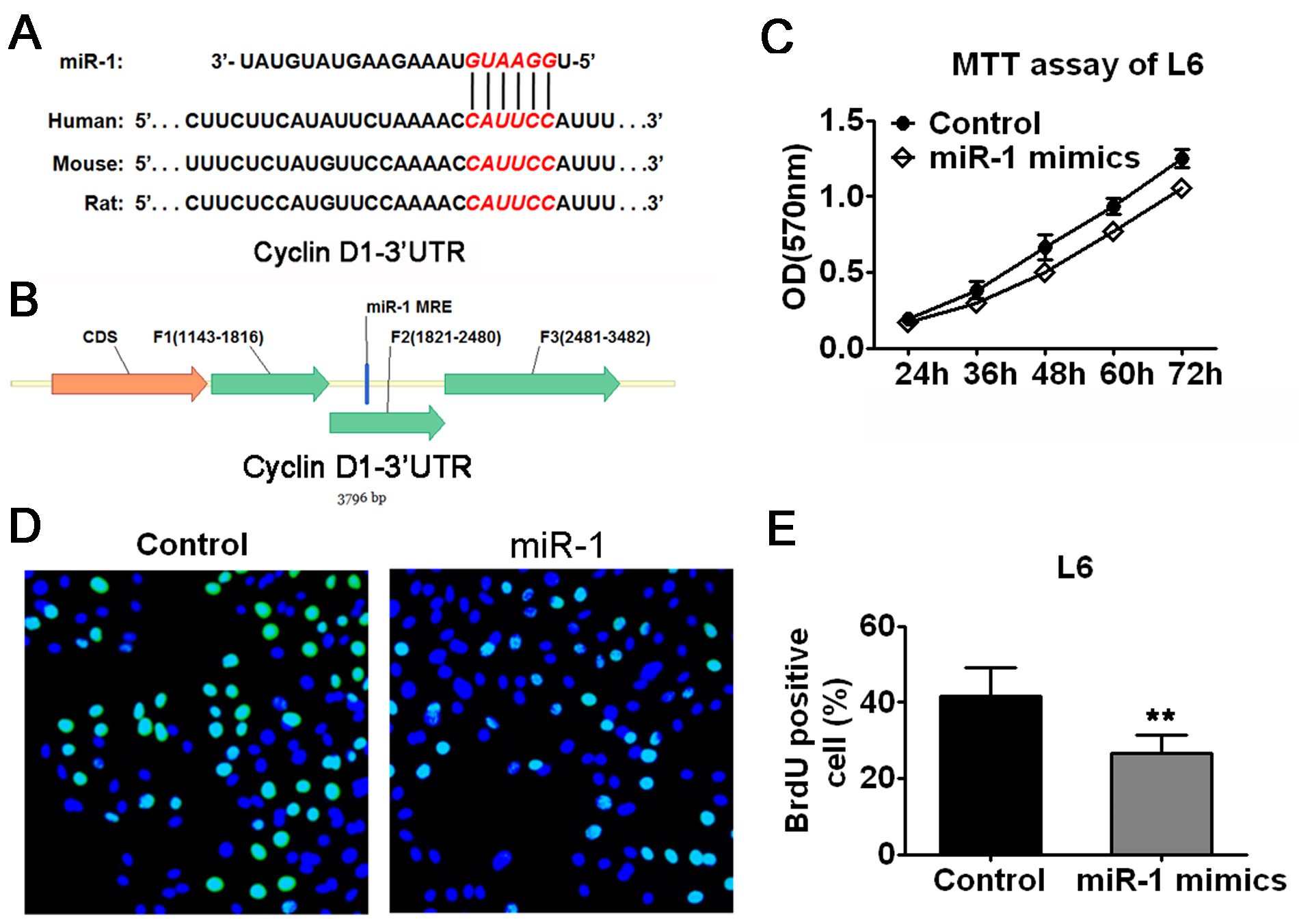

Supplement: Figure S4 — miR-1 inhibits myoblast proliferation via targeting Cyclin D1. (A) Comparison of miR-1 regulatory elements in the 3′UTR of Cyclin D1 across species. (B) Schematic representation of reporters containing three different regions of Cyclin D1–3′UTR, designated F1, F2 and F3. (C) Growth curves of L6 myoblasts were determined by MTT assay. Cells were transfected with miR-1 mimics or control oligos. Error bars represent the SD of three independent experiments. (D and E) Proliferation of L6 myoblasts was evaluated by BrdU incorporation. Cells were transfected with miR-1 mimics. Representative images of cells were taken by fluorescence microscope (D). The percentage of BrdU positive cells was measured (E). Data shown are from a typical experiment performed in triplicate. **p<0.01. (TIF) [file pone.0041478.s004.tif]
