# Supplementary material for: Attenuation of p38-Mediated miR-1/133 Expression Facilitates Myoblast Proliferation during the Early Stage of Muscle Regeneration
Source: PLoS One. 2012 Jul 24;7(7):e41478. doi: 10.1371/journal.pone.0041478 (PMC3404058; doi:10.1371/journal.pone.0041478)
Supplement: Table S1 — Oligos used in cloning. (DOC) [file pone.0041478.s005.doc]

| Table S1. Oligos used in cloning | | |
| --- | --- | --- |
| Name | Sequence(5’-3’) | Length(bp) |
| top strand 1 | CTAGAAAGCACAATGTGGATTTGGGGACCAAAGGTCAGGACAATGTGGATT | - |
| top strand 2 | TGTGGACCAATACTAAGAGAATATAACTTTTGTGGACCAATACTAAGAGC | - |
| bottom strand 1 | TTGGTCCACAAAAGTTATATTCTCTTAGTATTGGTCCACAAAAGTTATATTCACATT | - |
| bottom strand 2 | GGCCGCTCTTAGTATTGGTCCACAAAAGTTATATTCTCTTAGTA | - |
| miR-1-2 and miR-133a1 Enhancer-F | CGGGGTACCTCCGCCAAGAAGAAGCTAAA | 2533 |
| miR-1-2 and miR-133a1 Enhancer-R | CCGCTCGAGCATTGAAGAGGCGATTTGGT |
| miR-1-1 and miR-133a2 Enhancer-F | TCCCCCGGGCTGTGACCCTGGGAAGAT | 1900 |
| miR-1-1 and miR-133a2 Enhancer-R | CCGCTCGAGTTGATGAGGCCCTTCCTG |
| miR-206 and miR-133b promoter-F | TCCCCCGGGTCCTTCCTCTGCTCATATGG | 2534 |
| miR-206 and miR-133b promoter-R | CCGCTCGAGGCTTTCGGCTTGTTAGTAGT |
| SP1-3'UTR-F1-F | TGCTCTAGAGATTAGACACCCAGTGCCAG | 621 |
| SP1-3'UTR-F1-R | AAGGAAAAAAGCGGCCGCGGATGCCAGGACAACATTTA |
| SP1-3'UTR-F2-F | TGCTCTAGAGATTAGACACCCAGTGCCAG | 621 |
| SP1-3'UTR-F2-R | AAGGAAAAAAGCGGCCGCGGATGCCAGGACAACATTTA |
| CCND1-3'UTR-F1-F | TGCTCTAGAGCTTCGAGATGCTGAGGACT | 476 |
| CCND1-3'UTR-F1-R | AAGGAAAAAAGCGGCCGCTCAAAATTCCAGGCCAGTCT |
| CCND1-3'UTR-F2-F | TGCTCTAGAACCTGCAGGTTCACAGCACC | 660 |
| CCND1-3'UTR-F2-R | AAGGAAAAAAGCGGCCGCAGGGCAGAGGTGTGCGTTTG |
| CCND1-3'UTR-F3-F | TGCTCTAGAGCTGGAGAAGGTTTAGGGCC | 1002 |
| CCND1-3'UTR-F3-R | AAGGAAAAAAGCGGCCGCAACTTGGGGGCCTACAGCACTG |
| MDH1-133a-F | CCGGAATTCTCCAAATAAGGTTGACAGTTGC | 276 |
| MDH1-133a-R | CCGCTCGAGTGTGGGAACCTCTAATACCTGTC |
| MSCV-Sp1-F | CCGCTCGAGTTCGCTTGCCTCGTCAGC | 2496 |
| MSCV-Sp1-R | CCGGAATTCGTGGGTATTGGCCCATATGTC |
