# Supplementary material for: Attenuation of p38-Mediated miR-1/133 Expression Facilitates Myoblast Proliferation during the Early Stage of Muscle Regeneration
Source: PLoS One. 2012 Jul 24;7(7):e41478. doi: 10.1371/journal.pone.0041478 (PMC3404058; doi:10.1371/journal.pone.0041478)
Supplement: Table S2 — Primers used in qRT-PCR. (DOC) [file pone.0041478.s006.doc]

| Table S2. Primers used in qRT-PCR | | |
| --- | --- | --- |
| Name | Sequence(5’-3’) | Length(bp) |
| mGAPDH-F | ACATCATCCCTGCATCCACT | 224 |
| mGAPDH-R | GTCCTCAGTGTAGCCCAAG |
| hGAPDH-F | ATGGGGAAGGTGAAGGTCG | 108 |
| hGAPDH-R | GGGGTCATTGATGGCAACAATA |
| mSp1-F | GCCGCCTTTTCTCAGACTC | 131 |
| mSp1-R | TTGGGTGACTCAATTCTGCTG |
| FGF2-F | TTGTGTCTATCAAGGGAGTGTGT | 167 |
| FGF2-R | TGCCACATACCAACTGGAGTATT |
| mCcnd1-F | GCGTACCCTGACACCAATCTC | 183 |
| mCcnd1-R | CTCCTCTTCGCACTTCTGCTC |
| hCcnd1-F | GAACAAACAGATCATCCGCAAAC | 166 |
| hCcnd1-R | GCGGTAGTAGGACAGGAAGTTG |
| hSP1-F | GGTGCCTTTTCACAGGCTC | 130 |
| hSP1-R | CATTGGGTGACTCAATTCTGCT |
| U6 | ATTCGTGAAGCGTTCCATAT | - |
| miR-133 | TTTGGTCCCCTTCAACCAGC | - |
| miR-1 | TGGAATGTAAAGAAGTATGTAT | - |
| miR-206 | TGGAATGTAAGGAAGTGTGTGG | - |
| TNFa-F | AGCCGATGGGTTGTACCTTGTCTA |  |
| TNFa-R | TGAGATAGCAAATCGGCTGACGGT |  |
| IL-6-F | ATCCAGTTGCCTTCTTGGGACTGA |  |
| IL-6-R | TAAGCCTCCGACTTGTGAAGTGGT |  |
